# Therapeutic targeting of tumour-associated macrophage receptors

**DOI:** 10.1093/immadv/ltaf009

**Published:** 2025-03-11

**Authors:** Rosa Gomes Alves Martins, Mehmet M Tekin, Mark S Cragg, Ali Roghanian

**Affiliations:** Antibody & Vaccine Group, Centre for Cancer Immunology, School of Cancer Sciences, Faculty of Medicine, University of Southampton, Southampton General Hospital, Southampton, United Kingdom; Antibody & Vaccine Group, Centre for Cancer Immunology, School of Cancer Sciences, Faculty of Medicine, University of Southampton, Southampton General Hospital, Southampton, United Kingdom; Antibody & Vaccine Group, Centre for Cancer Immunology, School of Cancer Sciences, Faculty of Medicine, University of Southampton, Southampton General Hospital, Southampton, United Kingdom; Institute for Life Sciences, University of Southampton, Southampton, United Kingdom; Antibody & Vaccine Group, Centre for Cancer Immunology, School of Cancer Sciences, Faculty of Medicine, University of Southampton, Southampton General Hospital, Southampton, United Kingdom; Institute for Life Sciences, University of Southampton, Southampton, United Kingdom

**Keywords:** tumour-associated macrophages, tumour microenvironment, immunotherapy, monoclonal antibodies

## Abstract

Tumour-associated macrophages (TAM) are present in the majority of tumours, where they comprise one of the most abundant cell types, influencing tumour progression, metastasis, therapy resistance, and relapse. Hence, there is a great interest in targeting TAMs to improve and complement anti-cancer treatments. However, further studies are needed to validate the potential of exploiting TAM cell surface markers for cancer immunotherapy. Here, we review the function of TAMs, their involvement in tumorigenesis, metastasis, and therapy resistance. Furthermore, we summarize the current landscape of key TAM cell surface receptors that are being investigated as potential targets for cancer immunotherapy, highlighting the promise and challenges associated with these approaches.

## Introduction

### Macrophages

Myeloid cells constitute a major immune cell population in the tumour microenvironment (TME) and are able to regulate tumour growth by direct and/or indirect interactions with cancerous cells [1, 2]. Macrophages, in particular, are an important part of the myeloid mononuclear phagocytic system, residing in nearly all tissue types. They were the first leukocytes to be characterized and recognized for their involvement in the TME, the discovery of which saw Metchnikoff awarded the Nobel Prize in Physiology or Medicine in 1908 [3]. Macrophages perform diverse functions, and, as well as acting as the first line of defence against exogenous and endogenous threats, they maintain tissue homeostasis, regulating all phases of tissue healing and repair. In addition, they are involved in the initiation and resolution of inflammation and can present antigens to T cells [4].

Macrophages have two distinct origins: deriving from tissue-resident macrophages (TRM) or circulating bone marrow-derived monocytes. TRMs originate either from the yolk sac or foetal liver during embryonic development and are distinct from the circulating monocytes [5]. As a consequence of tissue-specific microenvironments, TRMs are heterogenous and exhibit tissue-specific morphologies and functions [6]. For example, Kupffer cells are specialized liver macrophages that are able to recognize and remove pathogens/debris from the blood as it circulates through the liver, via the scavenger and pattern recognition receptors they express [7]. TRMs also perform homeostatic functions within their respective tissues under normal, steady-state conditions, and can proliferate in situ [8, 9]. Indeed, alveolar macrophages, the major macrophage population in the lung, reside on the epithelial surface of alveoli where they are in direct contact with inhaled particulates and invading pathogens. They protect the air-exposed space of the alveolus by phagocytosing these targets and clearing mucus material from the alveolus, ensuring efficient gas exchange within the healthy lung [10]. Conversely, circulating bone marrow-derived monocytes are only found after inflammatory or pathological insults. They then co-exist with TRMs [6], sculpted by the local environment with most studies suggesting that the majority of macrophages, within any given tissue, originate from blood-borne monocytes [11]. Monocytes are recruited to inflamed or injured tissues via chemotactic gradients, and, upon arrival, acquire phenotypic characteristics driven by the nature of the differentiation signals present in that niche [12]. Within these tissues, macrophages can detect pathogen-associated molecular patterns (PAMPs) [13], such as bacterial products, and damage-associated molecular patterns (DAMPs) produced in response to trauma, ischaemia, or tissue damage [14]. This process utilizes a system of pathogen recognition receptors (PRR), such as toll-like receptors (TLR), which can specifically bind to pathogen components such as bacterial lipopolysaccharide (LPS), RNA, DNA, and other extracellular macromolecules, leading to the activation of various transcription factors, such as members of the signal transducer and activator of transcription family (STAT), nuclear factor-κB (NF-κB), or activator protein 1 [15, 16]. Depending on these environmental cues, macrophages may adopt certain functions and from these characteristics, researchers have attempted to classify macrophages based on defining features.

#### Macrophage classification

Macrophages are equipped to execute a broad repertoire of functions that range from mediating tissue homeostasis and wound healing to immune effector activities. To perform these different functions, macrophages have a high degree of plasticity; and thus, are able to flexibly adapt their metabolic, phenotypic and functional properties in response to microenvironmental cues in the their local environment [4]. Additionally, it allows them to balance the immune response such that an overwhelming pro-inflammatory response can be countered by an anti-inflammatory response [17]. Thus, macrophages were previously categorized into two groups with opposing functional characteristics: classically activated macrophages (M1), and alternatively activated macrophages (M2) [18]. Mills *et al*. termed them pro- and anti-inflammatory macrophages, “M1” and “M2”, respectively, as they mirrored the type 1 (Th1) and type 2 (Th2) polarization of CD4^+^ T helper cells observed in mice [19]. Pro-inflammatory cytokines such as interferon (IFN)-γ (secreted by Th1 cells and natural killer [NK] cells) and bacterial LPS serve as cues to trigger pro-inflammatory, anti-bacterial, anti- angiogenic, so-called “M1” functions and are used to produce classically activated macrophages *in vitro*. This results in the expression of key effector molecules by macrophages that allow for pathogen recognition and killing, as well as recruitment of other immune cells to the site of infection. Generation of reactive oxygen species (ROS) and nitric oxide (NO) and expression of pro-inflammatory factors including interleukin (IL)-1β, IL-6, IL-12, and tumour necrosis factor (TNF)-α, are typically associated with an “M1-like” macrophage response [20]. ROS and NO are fundamental for macrophage elimination of invasive micro-organisms and mediate protection against infection, respectively [21]. Conversely, secretion of cytokines, such as IL-4 and IL-13 by Th2 cells induce macrophages to adopt a more anti-inflammatory “M2- like” functional profile, resulting in the expression of anti-inflammatory factors, such as IL-10 and transforming growth factor (TGF)-β. Under physiological conditions, “M2-like” macrophages are thought to facilitate wound healing by inducing angiogenesis, cell proliferation, and clearing of cellular debris [20].

Although the M1 and M2 polarization and classification has revealed important details regarding macrophage function, the advent of new technologies, such as single-cell RNA sequencing (scRNA-seq), and spatial approaches, has shown that the distinction between these activation states is less clear [22, 23]. Macrophages can exhibit phenotypes that lie anywhere between these two extremes and are considered a highly heterogeneous group of immune cells [24]. Additionally, the M1/M2 classification was originally defined using murine macrophages [19] but M1 and M2 macrophage signatures found in mice are rarely recapitulated in their human counterparts [4]. Thus, the use of the “M1” and “M2” classification remains controversial, given the lack of tightly defined criteria to categorize phenotypes. However, efforts to more appropriately define macrophage signatures are continually advancing [18, 25].

**Figure 1.**
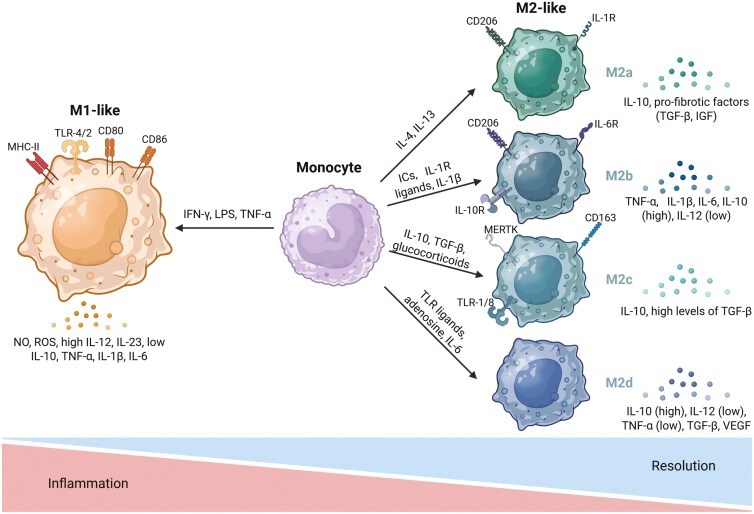
Macrophage polarization is dependent on different environmental cues. Stimuli within the microenvironment allows for differentiation of monocytes into different macrophage states with a particular array of functions. Although macrophages represent a continuum, they can be broadly divided into “M1-like” and “M2-like”, with the latter being further subdivided into M2a, M2b, M2c, and M2d phenotypes. All of these subsets express different cytokines, chemokines, and receptors allowing them to perform different functions. Created in BioRender. Martins, R. (2025) https://biorender.com/v67o359

Prior to this more sophisticated understanding of macrophage states, M2 macrophages were further divided into M2a, M2b, and M2c subclasses, according to a number of potentially relevant stimuli [26, 27] (**Table 1**). M2a or “wound-healing” macrophages, are generated upon exposure to IL-4 and IL-13, express high levels of the mannose receptor, CD206, and are proposed to mirror those involved in wound healing and fibrosis [28]. M2b or “regulatory” macrophages differentiate in response to a combination of immune complexes (IC) and TLR (e.g. LPS) and/or IL-1 receptor agonists. This subclass produces both anti- and pro- inflammatory cytokines, such as, IL-6, IL-1β, TNF-α, IL-10, and IL-12, regulating the breadth and depth of the immune response and the inflammatory reaction [29]. Finally, M2c macrophages are generated on exposure to a combination of glucocorticoids and IL-10-induced STAT3 activity, and exhibit strong anti-inflammatory activities mediated by high levels of TGF-β and IL-10 [29]. The production of these anti-inflammatory cytokines can dampen the pro-inflammatory effector functions of adaptive immune cells, mediating the resolution of inflammation (**Fig. 1**). The high expression of Mer receptor tyrosine kinase (MerTK) on M2c macrophages results in their efficient phagocytosis of apoptotic cells (efferocytosis) [30]. Additionally, M2d macrophages, proposed to be related to those common in the TME, belong to a more recently identified subtype of macrophages that are induced by TLR ligand and adenosine co-stimulation and/or IL-6 exposure. M2d macrophages express both VEGF and IL-10, reportedly participating in angiogenesis and tumour progression [20, 31]. Given that the differential activation of macrophages can promote or inhibit inflammation as well as regulate tumour proliferation, it is vital to further characterize them within tissues to better understand their specific functions and phenotype.

**Table 1.** M1 and M2 macrophages show distinctive biological and physiological features. Information below was derived from the following articles [20, 24, 29, 31].

Status	Stimuli	Signature markers	Secreted mediators	Functions
**M1**	IFN-γ, LPS, TNF-α	CD80, CD86, MHC-II	NO, ROS, high IL-12, IL-23, low IL-10, TNF-α	Pro-inflammatory Th1 response, anti-tumour
**M2a**	IL-4, IL-13	CD206, IL-1R	IL-10, pro-fibrotic factors such as TGF-β, insulin-like growth factor (IGF), and fibronectin.	Anti-inflammatory, wound healing/tissue repair
**M2b**	ICs, IL-1R ligands, IL-1β	CD206, IL-6R, IL-12R, IL-10R	TNF-α, IL-1b, IL-6, IL-10 (high), IL-12 (low)	Th2 activation (promoting infection), immunoregulation
**M2c**	IL-10, TGF-β, glucocorticoids	CD163, MerTK, TLR-1/8	IL-10, high levels of TGF-β	Efferocytosis, tissue remodelling, immunosuppression
**M2d**	TLR ligands, adenosine, IL-6	VEGF	IL-10 (high) IL-12 (low), TNF-α (low), TGF-β	Promotion of angiogenesis and tumour growth

#### Tumour-associated macrophages (TAM)

Macrophages that reside within the TME are typically referred to as TAMs and are among the most abundant immune population within it, comprising up to ~50% of the haematopoietic cells [2, 32]. Several meta-analyses have highlighted the correlation between high TAM frequency and poor overall survival in several cancer types [33], including breast, gastric, oral, ovarian, bladder, and thyroid cancer, though interestingly not in colorectal cancer [34–37]. Moreover, substantial clinical and experimental evidence indicates that macrophages have a major role in promoting tumour progression to malignancy [3, 38, 39]. Therefore, TAMs represent a major target for the development of new immunotherapies [2].

##### TAM origin

Consistent with the diverse ontogenies of macrophages in normal development and homeostasis, TAMs originate from both yolk sac-derived TRMs and newly recruited monocytes that differentiate within the TME. Studies in mice suggest that in pancreatic cancer and glioma, TAMs predominantly originate from the yolk sac and foetal liver [40, 41]. In a murine model of pancreatic ductal adenocarcinoma (PDAC), pancreatic resident macrophages were shown to be of embryonic origin locally proliferating in situ during tumour progression and behaving as pro-tumoural macrophages, whereas those originating from bone marrow displayed an antigen-presenting phenotype and potentially an anti-tumour phenotype [40]. In contrast, in a mouse model of lung cancer, lineage tracing experiments revealed that the pool of TAMs are initially composed of TRMs and only in the later phases of tumour development, are they dominated by monocyte-derived TAMs that have differentiated locally [42]. In mouse models of breast cancer, it was demonstrated that depleting C-C chemokine receptor type 2^+^ (CCR2) inflammatory monocytes resulted in the loss of 96% of tumour-associated monocytes together with ~93% of TAMs [43]. This suggests that mammary TRMs contribute minimally to the TAM pool compared to circulating inflammatory monocytes.

Whilst the differentiation and origin of murine macrophages in general, as well as TAMs, is generally better understood, the proliferation/origin of human macrophages remains poorly documented. A recent study in cancer patients undergoing bone marrow transplantation demonstrated the importance of monocyte recruitment in maintaining pools of TAMs [44]. This supports the dogma that the pool of TAMs in a fast-growing tissue likely result from the recruitment of monocytes [4]. However, the relative contribution of tissue-resident versus monocyte-derived TAMs in tumorigenesis appears tumour-type dependent and warrants further investigation.

TAM can be derived from blood monocytes that migrate into the tumour following a variety of chemotactic signals (mainly produced by tumour cells), such as macrophage colony-stimulating factor (M-CSF, CSF-1), CCR2, and VEGF [16]. There has been an interest in targeting the M-CSF/CD115 axis with small molecule inhibitors to reduce monocyte infiltration, macrophage differentiation, and survival and thus improve patient outcomes [45]. Another ligand-receptor pair known to be involved in monocyte recruitment and which has garnered interest as potential targets for immunotherapies is CCR2/C-C motif chemokine ligand 2 (CCL2; monocyte chemoattract protein-1 [MCP-1]). Recent studies have shown that therapeutic targeting of CCR2 results in tumour regression and sensitization to treatment (see below). This highlights that monocyte relocation to the TME and the factors that mediate this process, as well as their subsequent proliferation and differentiation into TAM, play a critical role in cancer aetiology and warrant further investigation.

##### Roles of TAMs in cancer

**Figure 2.**
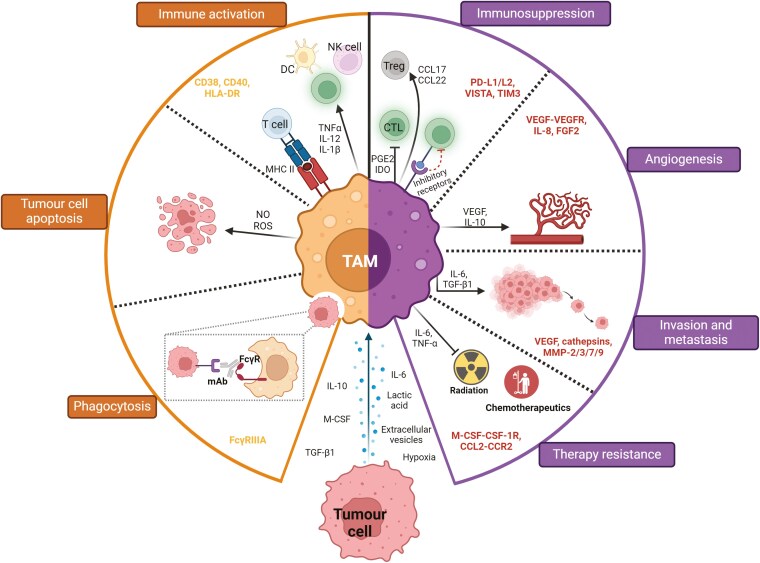
Anti-tumorigenic and pro-tumorigenic functions of TAMs. Tumour-educated macrophages can promote (right) or suppress (left) tumour development, depending on their activation status and environmental cues. Anti-tumour macrophages can induce cancer cell death via secretion of mediators or through direct cellular interactions. On the other hand, pro- tumour TAMs can mediate immunosuppression by secreting factors into the TME and expressing surface molecules that lead to recruitment of Tregs and inhibition of CTLs. In addition, TAMs have been linked to increased invasion and metastasis, angiogenesis/lymphoangiogensis, and resistance to radiotherapy and chemotherapeutics, as well as disease recurrence. Created in BioRender. Martins, R. (2025) https://biorender.com/s36p829

Monocyte-derived macrophages as well as TRMs can respond to the presence of tumour cells with local proliferation and differentiation into TAMs. The inherent ability of macrophages to adapt in response to alterations of microenvironmental stimuli means the range of phenotypes TAMs can adopt is highly diverse, as the tumour niche features an everchanging gradient of nutrients, metabolites, oxygen and other molecules [46–48]. Due to their diversity, TAMs feature a wide range of functionalities, ranging from tumour cell trophic support, invasion/metastasis promotion, therapy resistance, and suppression of anti-tumour immunity to supportive anti-tumour responses through T cell activation and tumour cell clearance [49] (**Fig. 2**). These opposing functions depend on many factors, including the disease stage and cancer type. Notably, macrophages can initially mount a robust anti-tumoural response, as they are able to directly eliminate cancer cells and support the adaptive immune response through presentation of tumour antigens and the production of chemokines and cytokines to recruit and activate cytotoxic T lymphocytes (CTL) and NK cells [50]. However, in solid tumours, the immunosuppressive TME can divert macrophage function from tumouricidal to trophic, with the latter function reminiscent of macrophages involved in tissue repair during inflammation, causing suppression of anti-tumour immunity [2].

As introduced above, for many years, the diversity of TAMs was underestimated, with the consensus being that TAMs have a polarization program resembling that of M2 macrophages [51]. Although active TAMs do share properties similar to those of M2a, M2c, and M2d macrophages (angiogenesis, remodelling, wound healing/tissue repair) (**Table 1**), progress in multiomics and particularly single cell technologies has revealed that they can adopt multiple distinct states based on gene expression profiles [16]. Indeed, as reviewed by Ma and colleagues, TAMs can acquire a broad spectrum of activation and functional states, and thus those authors proposed a new consensus model of TAM subsets that may help build a more complete understanding of the heterogenous and dynamic interactions between TAM subsets and tumour cells, as well as with the other constituents of the TME [38]. By reviewing several single cell multiomics studies of cancer, Ma *et al*. identified seven TAM subsets preserved in almost all cancer types and proposed terming these TAM subsets as interferon-primed, immune-regulatory, inflammatory cytokine-enriched, lipid-associated, pro-angiogenic, TRM-like TAMs and proliferating TAMs, depending on their predicted function, reinforcing that TAMs exhibit extraordinary plasticity [38]. Whilst TAM diversity ranges widely across various cancer types, each of which itself is highly heterogeneous, it is clear that the interaction of TAMs with cancer and stromal cells within the TME enables and sustains most of the hallmarks of cancer [33]. In addition to patient data [34–37], this has been corroborated by preclinical studies utilizing different murine tumour models, that demonstrate an association between macrophage depletion and reduced tumour progression and marked inhibition of metastasis [46].

TAMs secrete multiple factors that can support tumour growth and aid the epithelial- mesenchymal transition (EMT) of tumour cells, such as IL-1β, IL-16, and TGF-β, as well as extracellular matrix (ECM)-degrading proteins, such as cathepsins, matrix metalloproteinases (MMP7, MMP2, and MMP9), and serine proteases that enable tumour cell migration, via ECM remodelling [52–54]. TAMs also produce epidermal growth factor (EGF) receptor ligands, which enhance tumour motility and induce stem-cell-like properties [55]. Additionally, they release pro-angiogenic factors, such as VEGF and fibroblast growth factor 2 (FGF2), that not only increase tumour vascularization but also promote metastasis by enhancing tumour cell movement and intravasation [56]. Notably, in patients with non-small-cell lung cancer (NSCLC), it was demonstrated that high expression of VEGF in TAMs promotes angiogenesis and associates with poorer prognosis [57]. Moreover, studies using conditioned medium from TAMs and TAM/cancer cell line co-cultures have shown that TAMs promote cell migration and invasion of various human tumour cell lines via an MMP9-dependent mechanism [58, 59]. Furthermore, TAMs have been shown to promote EMT in hepatocellular carcinoma (HCC) cell lines via secretion of TGF-β [60], reinforcing the important role TAMs and the factors they secrete in tumour metastasis/invasion.

TAM-mediated immunosuppression can disrupt the immune-effector cell functions required for tumour clearance. Some of the known mechanisms by which TAMs promote and/or support an immunosuppressive TME include producing high levels of cytokines and chemokines including IL-10, TGF-β, and prostaglandin E2 (PGE2), all of which can inhibit the activation and function of CTLs and induce regulatory T (Treg) cell expansion [2]. Notably, on exposure to suppressive stimuli, such as CXCL8 (also known as IL-8) and PGE2, TAMs can upregulate immune checkpoint ligands, such as PD-L1/PD-L2, VISTA, and TIM3, which directly inhibit CTL functions, further contributing to immunosuppression [2, 61–63]. In addition, TAM production of chemokines, such as CCL2, CCL3, CCL4, and CCL20, lead to further Treg recruitment to the TME [2]. The immunosuppressive capacity of TAMs has been demonstrated both *in vitro*, whereby macrophages isolated from mouse and human tumours can directly suppress T cell responses, as well as *in vivo*, with depletion of TAMs leading to enhanced CD8^+^ T cell-mediated anti-tumour immunity [64, 65]. Given the above, immunotherapies targeting TAMs are currently being investigated as a method to alleviate TAM-mediated immunosuppression to enhance anti-tumour immune responses.

#### Current TAM-targeting strategies

**Figure 3.**
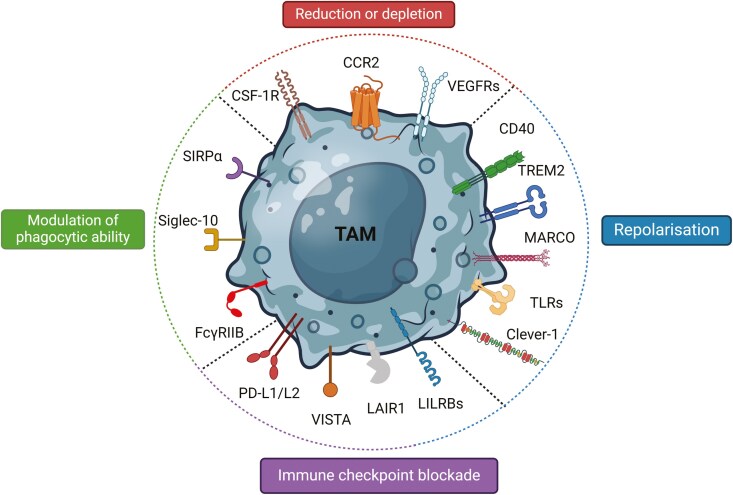
Current therapeutic strategies targeting TAM cell surface receptors. The main therapeutic strategies targeting TAM receptors include increasing phagocytic ability (impairing “don’t eat me” signal pathways), repolarization (from pro- to anti-tumour), reducing and decreasing survival and relocation, and immune checkpoint blockade with antibodies to relieve immunosuppression. The process of macrophage-mediated ADCP involves the recognition of tumour cells by therapeutic antibodies, such as rituximab (anti-CD20) and trastuzumab (anti- HER2) which then engage FcγRs on macrophages. Antibodies targeting “don’t eat me” receptors on macrophages (*e.g.*, SIRPα and Siglec-10) or target cells can enhance ADCP and improve immunotherapy responses. Created in BioRender. Martins, R. (2025) https://biorender.com/h79n993

As detailed above, the proportion of macrophages in solid tumours is generally associated with poor prognosis and correlates with therapy resistance in most cancers [3, 33]. Their multiple pro-tumoural activities make TAMs appealing targets for anti-cancer therapy and, thus, therapeutic strategies, including reduction or depletion of TAMs, TAM repolarization, and specific molecular targeting, have been proposed [11, 47]. A number of current approaches and TAM cell surface targets are highlighted in **Fig. 3**.

##### Reducing or depleting TAMs

The depletion of pro-tumoural TAMs represents a possible therapeutic approach for limiting tumour progression. Since macrophages relocate to tumours by tumour- and stroma-derived chemo-attractants, preventing these processes via pharmacological modulation may be an effective treatment modality for inhibiting the pro-tumour functions of TAMs. However, this strategy might be limited, as it indiscriminately depletes macrophages, potentially affecting monocyte/macrophage-mediated host defence and homeostatic functions [66]. Despite these challenges, promising strategies have emerged. For example, liposomal clodronate (bisphosphonate) treatment, which relies upon the preferential uptake of liposomes by macrophages via phagocytosis, has been shown to reduce tumour infiltration and thereby attenuate lung cancer progression *in vivo* [67]. In addition, trabectedin (a DNA alkylating agent), originally approved/developed as an anti-proliferative agent for soft tissue sarcoma and relapsed ovarian cancer was reported to induce apoptosis of intra-tumoural monocytes and macrophages through caspase 8 activation via a TNF-related apoptosis-inducing ligand (TRAIL)-dependent mechanism [66]. Moreover, given the dependency of macrophages on M- CSF/CSF-1R signalling to survive and/or proliferate within the TME, targeting this pathway was of particular interest previously (see below).

As mentioned in the previous section, targeting the CCR2/CCL2 signalling axis as well as VEGFR2 can reduce macrophage infiltration and suppress tumour growth in murine models [68–70]. For example, a recent study reported that CCR2 blockade could enhance the efficacy of anti-PD-1 therapy in *in vivo* lung and mammary tumour models by reducing macrophage frequency in tumours; thereby reducing Treg expansion and increasing effector T cell infiltration [68, 69]. Based on the shortcomings of these approaches, alternative therapeutic strategies to target TAMs including reprogramming have been proposed which could allow for more focussed targeting and a reduced likelihood of toxicity. Additionally, molecular targeting has emerged as a promising direction for selectively depleting immunosuppressive TAMs. For example, Cieslewicz and colleagues constructed an M2-macrophage targeting fusion peptide to selectively deplete pro-tumour TAMs or more M2-like macrophages, thereby reducing systemic damage [71]. Similarly, the expression of CD163 by TAMs is a strong indicator of poor prognosis in several cancers. Depletion of CD163^+^ TAMs by the Lawrence group in experimental models promoted anti-tumour immunity through activation of CTLs [72, 73]. Below is a list of key targets that have been investigated for reducing/depleting TAMs in cancer patients.

###### CSF-1R

CSF-1R plays important roles in innate immunity by regulating macrophage survival, proliferation, and differentiation. It exerts its biological functions as a transmembrane glycoprotein in response to two competing ligands, namely colony-stimulating factor-1 (CSF- 1) and IL-34. CSF-1R belongs to the type III protein tyrosine kinase receptor family, and binding of CSF-1 or the more recently identified ligand, IL-34, induces homodimerization of the receptor and its subsequent activation [8]. CSF-1R mediated signalling, upon ligation by M-CSF, is crucial for the differentiation and survival of the mononuclear phagocyte system and macrophages in particular [7]. In NSCLC, the degree of monocyte infiltration is associated with the expression of M-CSF in the tumour stroma and correlates with decreased overall and disease-free survival rates [74]. Furthermore, in glioma patients, high M-CSF expression significantly correlates with higher-grade glioma [75]. These data demonstrate that M-CSF overexpression in tumours is associated with disease progression potentially via monocyte recruitment. Furthermore, M-CSF not only stimulates monocyte infiltration but also induces their differentiation/maturation into macrophages and subsequently regulates macrophage survival and proliferation [76].

As the intratumoural presence of CSF-1R^+^ macrophages correlates with poor survival in various tumour types [5, 9], targeting CSF-1R signalling in TAMs represents an attractive strategy to eliminate or repolarize these cells. A CSF-1R blocking antibody monotherapy achieved modest tumour growth inhibition by reducing TAMs and improving the ratio of CD8^+^ T cells to Tregs in colon adenocarcinoma and melanoma syngeneic tumour models [77]. In murine PDAC the anti-tumour efficacy of M-CSF/CSF-1R blockers was strengthened when combined with immune checkpoint inhibitors, as tumour progression was reduced by >90% [78]. Experimental studies also demonstrated that BLZ945, a highly selective small molecule CSF-1R inhibitor, not only inhibits monocyte recruitment into murine breast cancer by 6-fold but may also increase the infiltration of CTLs in cervical and breast carcinomas, relieving immunosuppression [11, 79]. Clinically, while kinase inhibitors like imatinib have shown limited success in inhibiting CSF-1R function, monoclonal antibodies (mAb) such as RG7155 have demonstrated significant efficacy in reducing TAMs and stabilizing disease in various cancers, highlighting CSF-1R as a promising therapeutic target [80]. Moreover, blockade of CSF-1R by AMG 820 was shown to reduce the accumulation of immunosuppressive TAMs in solid tumours by ~60% [81]. Despite these promising results, initial clinical trials have been underwhelming, with some TAM subsets apparently resistant to this approach [82, 83]. Additionally, M-CSF/CSF-1R blockers cause severe toxicity in multiple organs and tissues as M-CSF is essential for maintaining normal macrophages for inflammation resolution and pathogen elimination [84]. Nevertheless, there is currently one clinically approved small molecule CSF-1R inhibitor, PLX3397 (pexidartinib), showing efficacy for the treatment of tenosynovial giant cell tumours, with combination therapies and innovative molecular targeting strategies showing promise in other settings [85].

###### CCR2

CCR2 is constitutively produced under normal physiological conditions, facilitating lymphocyte trafficking and monocyte/macrophage mobilization. A central role of CCR2 in TAM accumulation is supported by data showing high levels of tumour-derived CCR2 concurrent with leukocyte recruitment in several cancer types, including mammary [39], pancreatic [86], and prostate carcinoma [87]. The importance of CCR2 within the TME was highlighted using a HCC model, where blocking the CCR2/CCL2 axis prevented the recruitment and infiltration of monocytes [88]. Furthermore, combination of CCR2 antagonism with anti-PD-1 therapy has been shown to lead to sensitization and enhanced tumour reduction in comparison to anti-PD-1 monotherapy [68].

CCL2 expressed by various stromal and immune cells, including endothelial, epithelial, and myeloid cells, as well as brain cells, acts as a potent chemoattractant by binding to its primary receptor, CCR2 [89]. CCL2-mediated recruitment of circulatory monocytes and monocytic myeloid-derived suppressor cells (MDSC) to tumour tissues leads to an abundance of TAMs, often associated with poor clinical outcomes, as evidenced by its correlation with TAM accumulation, early relapse, and vessel invasion in human breast cancer and poor prognosis in oesophageal carcinogenesis. High levels of CCL2 expression in other cancers are also associated with poor prognoses, such as lung adenocarcinoma and liver cancers [88–91]. The CCL2/CCR2 axis regulates macrophage polarization, since blocking CCL2 led to an upregulation of M1 polarization-associated genes and decreased expression of M2-associated markers in human macrophages [92]. CCL2 plays a role in tumorigenesis and metastases in several solid tumours, including breast cancer, melanoma, and prostate cancer [93, 94]. CCL2 blockade significantly slowed primary tumour growth and inhibited lung metastases in NSCLC models by altering TAMs to a more anti-tumour phenotype and activating CTLs. These anti-tumour effects were lost in immunodeficient mice or after CD8^+^ T cell depletion, highlighting the importance of these cells in the therapeutic response. Similarly, genetic deletion or blockade of CCL2/CCR2 inhibits primary liver tumour and metastatic growth leading to prolonged survival [88].

A number of clinical trials have investigated therapeutic targeting of the CCR2/CCL2 axis, with encouraging results reported in a number of settings, including PDAC and metastatic liver cancer [95, 96].

##### TAM Repolarization

Repolarization strategies represent a promising avenue to reprogram TAMs from an immunosuppressive state to an immunostimulatory one. This strategy leverages the inherent plasticity of TAMs to shift the TME from supporting tumour growth to inhibiting it. Despite being generally considered pro-tumoural, TAMs can, depending on context, be tumouricidal as well, suppressing tumour growth by promoting anti-tumour immunity (**Fig. 2**). This indicates that exploiting TAM plasticity to restore their anti-tumour properties is a promising therapeutic strategy, especially to enhance other cancer immunotherapies [2]. Unlike depletion strategies, TAM reprograming more selectively harnesses macrophage plasticity to modulate the TME. Thus, several studies have explored stimulating or blocking a diverse range of macrophage receptors or signalling pathways to drive immunosuppressive TAMs to adopt a more tumour suppressive role. For example, agonism or activation of TLRs has been shown to drive TAM reprogramming or repolarization towards a pro-inflammatory phenotype. Thus, several synthetic TLR ligands have been tested in different cancer models to assess their efficacy in switching TAMs from pro- to anti-tumour in the TME [2]. In an orthotropic mammary tumour mouse model, it was reported that TLR7 and TLR9 agonists caused increased monocyte infiltration into the tumour and macrophage repolarization [97]. Moreover, it has been shown that TLR3 stimulation can switch macrophages from an anti- to a pro- inflammatory phenotype/profile, leading to tumour regression [98], indicating that signalling through TLR3 can regulate TAM phenotype and possibly function and warrants further investigation. In addition, a number of cell surface therapeutic targets amenable to antibody therapy have been investigated for this purpose, as discussed below.

###### CD40

The CD40 receptor and its ligand, CD40L, are one of the key molecular pairs responsible for immune stimulation of both B cells and T cells. CD40 is a transmembrane glycoprotein cell surface receptor with a molecular weight of 48 kDa. CD40 expression is relatively high on B cells and myeloid cells, whereas CD40L is expressed highly on T cells and platelets [99]. Analysis of a human lung scRNA-seq dataset supported that high levels of CD40 are present on myeloid and B cells and elevated levels of CD40L are evident on T cells [99].

The CD40-CD40L interactions play a role in the licensing and activation of dendritic cells (DCs), leading to the activation of CTLs [100]. Hence, the use of immunomodulatory agonistic mAbs targeting CD40, a T cell costimulatory ligand expressed by antigen-presenting cells (APC), including macrophages, is of great interest. TAMs activated by CD40 show enhanced antigen presentation and T cell costimulation, associated with increased MHC class II and CD86 expression, and have been shown to promote tumour regression in a murine model of PDAC [101, 102]. CD40 agonism can also reprogramme TAMs and stimulate the development of anti-tumour myeloid cells [101]. Whilst effective in preclinical models, results in the clinic have been disappointing as CD40 agonists have only resulted in partial responses in several clinical trials [103], likely due to either insufficient agonism or dose-limiting toxicities, depending on the reagent [104]. These clinical trial results warrant re-thinking the strategy, for example by re-engineering the CD40 mAbs, evaluating bispecific CD40 antibodies, as well as providing localized and sustained delivery of anti-CD40 agonists (where possible) alongside combination therapies.

###### Macrophage receptor with collagenous structure (MARCO)

MARCO is a member of the class A scavenger receptor family with a molecular weight of ~52 kDa. It exists as a trimeric receptor and is expressed on subsets of macrophages and TAMs [41]. In physiological conditions MARCO expression is specific to certain macrophage subsets, typically found in the spleen, lymph nodes, and peritoneum [105]. However, upon encountering pathogens like bacteria or viruses, MARCO can also be expressed on macrophages across various tissues, including the lungs [106, 107]. MARCO plays a crucial role in triggering the immune response, bridging innate and adaptive immunity, and eliminating pathogens. *In vitro* studies have shown that antibodies to MARCO promote glycolysis and exhibit the ability to inhibit tumour growth and metastasis in experimental breast cancer and melanoma models [108]. MARCO is increased on immunosuppressive TAMs in NSCLC, and if inhibited has been shown to repolarize TAMs facilitating the recovery of cytolytic activity, anti-tumour mediated immunity, and downregulation of Treg activity [109]. MARCO expression in human TAMs correlates with the expression of other checkpoint molecules. Accordingly, the combination of anti-CTLA-4 and anti-MARCO has been shown to increase the efficacy of melanoma and colon cancer treatment [108]. Blocking MARCO in a syngeneic melanoma tumour model also reduces the suppressive effects of TAMs on NK cells and enhances the efficacy of T cell-focussed immunotherapies, such as PD-1/PD-L1 inhibitors [110]. Similarly, a recent study reported a significant negative correlation between MARCO^+^ TAMs and the prognosis of patients with liver cancer [111]. Treatment of HCC-bearing mice with a blocking MARCO mAb significantly improved responses to anti-PD-L1 in HCC. This was shown to be via increased secretion of type I IFN by TAMs, leading to higher antigen presentation and tumour infiltration of CD8^+^ T cells. Taken together, these studies provide convincing evidence supporting MARCO as an attractive target for macrophage reprogramming, and the use of MARCO antibody immunotherapy shows promise for immunosuppressed cancers. Despite this potential, clinical trials involving MARCO antibodies have not yet begun, likely due to challenges in lead mAb optimization and safety profiling. However, an Investigative New Drug (IND) approval for MARCO antibodies is being sought [112].

###### Common lymphatic endothelial and vascular endothelial receptor 1 (Clever-1)

Clever-1 (Stabilin-1, FEEL-1) is a large glycoprotein receptor expressed on the surface of lymphatic endothelial cells, sinusoidal endothelial cells, and immunosuppressive macrophages and monocytes. Clever-1 is involved in scavenging, angiogenesis and cell adhesion. Genetic deficiency of macrophage Clever-1 reduces solid tumour growth by promoting an immunostimulatory TME and activating anti-tumour CD8^+^ T cells [113]. These effects are similar to those observed with PD-1 checkpoint inhibition. Combining anti-Clever- 1 with anti-PD-1 demonstrated synergistic benefits, particularly in aggressive, treatment- resistant tumours [113]. Studies with Clever-1-deficient mice have shown reduced tumour size and less metastasis. Tumour growth was also reduced when Clever-1 was absent in macrophages or vascular endothelium, though metastases was unaffected, and treatment with a Clever-1 blocking antibody inhibited tumour progression *in vivo* [114]. The deletion of functional Clever-1 genetically, or its blockade therapeutically, decreased the number of immunosuppressive TAMs and Treg in tumours [114]. Human cancer cohort analyses have linked the higher Clever-1^+^ macrophages with worst overall survival [115]. Further research in both mice and humans has shown that blocking Clever-1 can activate T cell responses by reprogramming macrophages and monocytes from immunosuppressive to pro-inflammatory states. Recent data mining across cancer cohorts revealed a significant correlation between Clever-1 expression and resistance to immune checkpoint therapies [116, 117]. Hence, Clever-1 is emerging as a potential therapeutic target to enhance immunotherapy efficacy and overcome resistance mechanisms in cancer treatment. As such, there is currently one recruiting clinical trial examining the tolerability, safety and preliminary efficacy of FP-1305, which is a humanized Clever-1 IgG4 mAb in patients with advanced, treatment-resistant cancers [118].

###### Triggering receptor expressed on myeloid cells-2 (TREM2)

TREM2 is an activating receptor of the Ig-superfamily, binding to lipids and transmitting signals via DAP12, which recruits the protein tyrosine kinase Syk to initiate downstream signalling cascades [119]. Interestingly, TREM2 can be cleaved from cell surfaces by MMPs to produce soluble TREM2, which may have regulatory effects on cell activation and promote cell survival in the TME [120]. The role of TREM2 in cancer is complex and context dependent. Studies have shown conflicting roles for TREM2 in different cancers; while TREM2 is protective in HCC, it correlates with poor survival in colorectal carcinoma and triple-negative breast cancer (TNBC) [121, 122]. A recent study investigating the impact of TREM2-expressing TAMs in NSCLC underscored the critical role of TREM2^+^ TAMs in driving immunosuppression, highlighting TREM2^+^ TAMs as potential prognostic markers and predictors of response to immunotherapy [123]. Additionally, TREM2^+^ TAMs have been shown to result in a reduction in infiltration of NK cells to the NSCLC TME, that can be reversed by blocking mAbs [124]. These studies shed light on the multifaceted role of TREM2 in cancer biology, emphasizing its potential as a therapeutic target and diagnostic marker in NSCLC and possibly other cancer types. A Phase 1 clinical trial investigated the efficacy of PY314, a TREM2-targeting mAb, and pembrolizumab. The combination was found to be safe but showed limited anti-tumour efficacy in patients with checkpoint inhibitor-refractory metastatic renal cell carcinoma (RCC), suggesting further research is needed to assess its potential in treatment-naïve settings (NCT04691375) [125]. A major limitation in the field is a clear understanding of the mechanism of action of TREM2 mAbs, and whether Fc engagement is required for the anti-tumour activity or not. With advancement in preclinical research, it is hoped more refined and efficacious therapeutic agents can be designed against human TREM2.

##### Immune checkpoint blockade

Over the last two decades immune checkpoint blockade has been one of the most promising approaches in cancer immunotherapy by reactivating the body’s immune cells against immune-suppressive cancer cells. In normal physiological conditions, immune checkpoints are regulatory pathways that play an important role in the homeostasis of the immune response and protect healthy tissues from excessive damage. However, tumours can subvert these checkpoints to suppress the destructive effect of cytotoxic immune cells. By targeting checkpoint receptors, such as PD-1/PD-L1, LAG-3, and CTLA-4 with specific antibodies, immune checkpoint blockade allows T cells to remain active and attack cancer cells more effectively. However, despite the success of T cell immune checkpoint inhibitors, many patients fail to respond or develop resistance overtime [126]. A critical mechanism underlying this resistance is TAM-mediated immunosuppression, making myeloid checkpoint targeting a promising area of research for overcoming these challenges. A number of promising myeloid checkpoint targets are discussed below.

###### V-domain Ig suppressor of T cell activation (VISTA)

VISTA (c10orf54, VSIR, SISP1, B7-H5, PD-1H, DD1α, Gi24, Dies1) is a type I transmembrane protein and consists of an N-terminal signal peptide, single IgV domain, transmembrane region, and cytoplasmic domain [127]. VISTA is expressed in both mice and humans, mainly on myeloid and granulocytic cells and relatively less on T cells [128]. VISTA shares 24% sequence similarity with PD‐L1 [129]. A study showed that immobilized VISTA- Fc suppressed the proliferation of anti-CD3 stimulated CD4^+^ and CD8^+^ murine and human T cells [130]. Furthermore, administration of a VISTA blocking antibody in allogenic mice prevented tissue rejection of naïve donor T cells and bone marrow cells [127]. VISTA has been shown to be ectopically expressed on certain human tumour cells, such as a subset of melanoma, colon, breast and PDAC cells, and its expression negatively impacts survival [131–138]. This ectopic expression has been shown to be associated with increased intratumoural Tregs and enhanced PD-L1 expression on TAMs [135, 139, 140]. VISTA binds to V-set and Ig domain-containing 3 and P-selectin glycoprotein ligand 1 ligands to elicit signalling that may be bidirectional [141]. Due to its immunoregulatory functions (extensively reviewed in [141, 142]), VISTA has been proposed to be a novel immune checkpoint receptor.

VISTA (*VSIR*) KO mice are prone to developing spontaneous autoimmunity, such as rheumatoid arthritis [143, 144]. Hypoxia-inducible factor 1α (HIF-1α), which is typically upregulated in the TME [145], has been shown to upregulate VISTA expression through hypoxia response elements in the *VSIR* promoter [131]. *VSIR* KO macrophages are more sensitive to pro-inflammatory mediators, such as TLRs [140, 146]. Mouse and human MDSCs express high levels of VISTA, which is believed to potentiate their immunosuppressive function [131, 137, 147, 148]. Together, these observations strongly suggest that blocking VISTA may provide a viable therapy against cancer by reprograming suppressive immune cells, such as MDSCs and TAMs. Indeed, VISTA blocking mAbs can limit the suppression of T cells in culture and exacerbate the development of a T cell-mediated autoimmune disease, experimental autoimmune encephalomyelitis, in mice [129]. Conversely, blockade of VISTA has been shown to provide cancer therapy in a number of experimental settings, either as single agent or in combination [140, 141, 149, 150]. Based on these positive responses, a number of clinical trials using VISTA antagonistic mAbs are currently underway [151–153].

###### PD-L1

PD-1 and its ligand PD-L1, also known as B7 homolog 1 (PD-L1, B7-H1, CD274), represent one of the most studied immune checkpoints in cancer and autoimmune disease. PD-L1 was discovered in 1999 [154]. Shortly after its initial description, several groups made important contributions to the knowledge we have about the engagement of PD-L1 with PD-1 and how it inhibits the proliferation and cytokine production of activated T cells [155–157]. In addition to resting B cells, PD-L1 is expressed on T cells, APCs and on the surface of many murine tumour cell lines [158]. In normal physiological conditions, PD-L1 expression on macrophages helps maintain inflammation homeostasis by interacting with PD-1 on activated T cells, leading to T cell suppression. This is crucial in preventing over reactive T cells. Macrophage PD-L1 expression plays a significant role in the resolution of inflammation [125]. The PD-L1/PD-1 interactions play an important role in regulating the activity of effector T cells, thereby preventing prolonged or excessive inflammation, which could lead to tissue damage or chronic inflammatory diseases. However, tumour cells or TAMs can manipulate CTLs via PD1-PDL1 interaction, ultimately leading to immune evasion. By overexpressing the immune checkpoint molecules (such as PD-L1), tumour cells are able to avoid the surveillance and destructive effects of immune cells in the TME, thus accelerating tumour development and metastasis in different tissues [159]. PD-L1 expression varies across tumour types and has been reported to be higher on NSCLC than RCC and have the lowest expression in melanoma, in both human tumour specimens and cell lines [160]. Although specific blocking mAbs against PD-1 and PD-L1 have made significant impacts clinically, insufficient infiltration levels of effector T cells in some solid tumour types or different expression levels of PD-L1 in tumour tissues may negatively affect the treatment efficiency of these immune checkpoint blockers [161, 162]. Undesirable toxic results can also be caused by excessive activation of T cells and is one of the limiting effects of PD-1/PD-L1 blocking therapies [163]. Since blockade of PD- 1/PD-L1 has shown limited ability to treat some of the tumours, ongoing clinical trials are mostly using PD-1/PD-L1 blocking mAbs with other myeloid targets covered in this review.

###### Leukocyte-associated Ig-like receptor-1 (LAIR-1)

LAIR-1 is a co-inhibitory receptor, with 2 immunoreceptor tyrosine-based inhibitory motif (ITIM) domains, expressed on several subsets of immune cells, and functions to delimit immune responses [164]. It binds to collagen and molecules with collagen-like domains [165, 166]. Several epithelial tumours, including breast, pancreatic, colorectal, ovarian, and lung cancer, are characterized by a dense ECM where high collagen content correlates with poor prognosis [167]. TME-expressed collagens promote immune evasion through direct interaction with LAIR-1 [168]. In cancer, it is hypothesized that LAIR-1 expression on several subsets of leukocytes, including myeloid cells and TAMs, prevents optimal immune responses by limiting both innate and adaptive immunity. NGM438, a LAIR1 mAb, is currently in a Phase 1/1b trial (NCT05311618) for the treatment of collagen-rich solid tumours.

###### Inhibitory leukocyte Ig-like receptors (LILRB)

**Figure 4.**
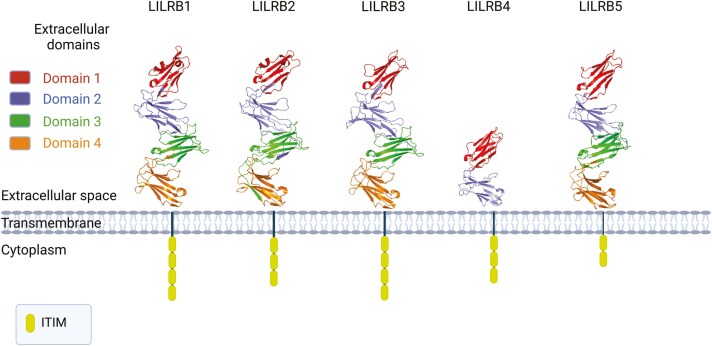
Inhibitory leukocyte immunoglobulin-like receptors. LILRs consist of 5 membrane-bound and one soluble LILRB. Of these receptors, LILRB1, LILRB2, LILRB3 and LILRB5 each contain 4 Ig-like extracellular domains, while LILRB4 contains 2 extracellular Ig-like domains. While LILRB1-5 contains 4, 3, 4, 3 and 2 ITIM domains intracellularly, respectively, LILRB2S is not membrane bound and lacks intracellular ITIMs. Created in BioRender. Martins, R. (2025) https://biorender.com/a18o606

Human LILRs (also known as LIRs, ILTs, and CD85) consist of eleven members, expressed on B, myeloid, NK and T cells [169, 170]. These receptors are divided into two main groups, six activating LILRs (LILRA1–6) and five inhibitory LILRs (LILRB1–5; **Fig. 4** and **Table 2**). Human LILRB genes are encoded by the leukocyte receptor complex located on the q13.4 region of chromosome 19 [187]. Immune regulatory functions of the LILRB family receptors can play a significant role in TME. For instance, LILRB1 and LILRB2 are involved in immune evasion by inhibiting T cell activation and promoting immune tolerance. Their interaction with HLA/MHC molecules on tumour cells and APCs contributes to the suppression of anti-tumour immunity, making them potential targets for therapeutic strategies aimed at enhancing immune responses against cancer (also see [188] for a recent comprehensive review).

**Table 2.** Summary of LILRBs, expression pattern and ligands.

Receptor	Other nomenclature	Expression	Number of ITIM domains	Number of Ig-like domains	Ligands
**LILRB1**	LIR1, ILT2, CD85j	Basophils, B cells, DCs, Eosinophils, Macrophages, Mast cell progenitors, Monocytes, NK cells, Osteoclasts, T cells	4	4	HLA-I [171] UL18 [172, 173] S100A8/9 [174]
**LILRB2**	LIR2, ILT4, CD85d	Basophils, DCs, Endothelial cells, Hematopoietic stem cells, Macrophages, Monocytes, Mast cell progenitors, Neutrophils, Osteoclasts, Platelets	3	4	ANGPTL [175] CD1c/d [176] CSP [177] HLA-I [178] MAG [179] Nogo66 [179] Omgp [179] SEMA4A [180] β-Amyloid [181]
**LILRB3**	LIR3, ILT5, CD85a	Basophils, Eosinophils, Mast cell progenitors, Monocytes, Neutrophils, Osteoclasts	4	4	S. aureus [182], Ligand(s) associated with cytokeratin8 [183] Galectin 4 and 7 [184]
**LILRB4**	LIR5, ILT3, CD85k	DCs, Endothelial cells, Macrophages, Mast cell progenitors, Monocytes, Osteoclasts, Plasmablasts, Tregs	2	2	ApoE [185] CD166 [186]
**LILRB5**	LIR8, CD85c	Mast cell granules, Monocytes, NKs, Osteoclasts, T cells	4	4	HLA-I [178]

####### LILRB1

LILRB1 (CD85j, ILT2, LIR1, or MIR7) has a relatively wide expression on human leukocytes and non-immune cells, including NK cells, placental stromal cells, granulocytes, monocytes/macrophages, eosinophils and basophils, DCs, subsets of T cells, B cell, immature mast cells, and osteoclasts. LILRB1 consists of four Ig-like domains extracellularly and 4 ITIM domains intracellularly and binds to β2m-associated histocompatibility antigen (HLA) class I molecules such as HLA-A, HLA-B, HLA-C, HLA-E, HLA-F and HLA-G, HLA class 1 homologue UL18, and a non-HLA class I calcium-binding protein, as well as S100A8/S100A9 ligands [174]. LILRB1 recognizes a wide variety of HLA haplotypes due to its interaction with the invariant β2M chain, which controls the phagocytic function of macrophages [189]. The HLA class I/LILRB1 signalling axis was identified as a negative regulator of macrophage effector function [189]. Continuous ligation of LILRB1 on human DCs hampers their T cell stimulatory capacity and promotes the development of tolerogenic DCs [190]. LILRB1 antagonism may therefore reactivate the tumour-supressed immune effector cells such as macrophages and CTLs. Several clinical trials are currently investigating therapeutic targeting of LILRB1, LILRB2, and LILRB4 (reviewed in [188]).

####### LILRB2

LILRB2 (CD85d, ILT4, LIR2, and MIR10) is structurally composed of 4 extracellular Ig-like domains connected with a transmembrane domain and 3 intracellular ITIM domains. LILRB2 is expressed by myelomonocytic cells, macrophages, and DCs and binds to both classical and non-classical class I molecules such as HLA-G, mediating a negative signal that recruits SHP-1 protein tyrosine phosphatase and inhibits early signalling processes on these cells [191]. LILRB2 is a distinct HLA class I receptor associated with the repression or down- modulation of monocyte activation signals by inhibiting Fc receptor-mediated signalling [192].

LILRB2 blocking in human primary lung cancer isolated macrophages reprogrammed TAMs to the classically activated phenotype [193]. It has been shown that in Lewis lung carcinoma- bearing LILRB2 transgenic mice, PD-L1 blockade had no effect on tumour growth, as monotherapy, whereas, LILRB2 blockade showed moderate impact, further enhanced by its combination with anti-PD-L1 [194]. Similarly, the combination therapy was more effective at reducing the tumour burden against human NSCLC (A549) cells in vivo, which was due to reprograming of human myeloid cells by anti-LILRB2 [194].

####### LILRB3

LILRB3 (CD85a, ILT5, LIR3, or HL9) consists of 4 extracellular Ig-like domains connected to 4 ITIM domains intracellularly, separated by a transmembrane domain. LILRB3 is expressed by monocytes, monocyte-derived osteoclasts, neutrophils, eosinophils, basophils, and osteoclasts [195]. Despite the elucidation of the key regulatory roles of many of the LILR family members in the immune system, our knowledge about LILRB3’s ligands is limited, and less is known about the immunoregulatory functions of LILRB3. Even though LILRB3 was previously described as an orphan receptor, it has recently been shown to interact with cytokeratin 8-associated ligands, upregulated on necrotic epithelial-derived tumour cells [183]. Moreover, a recent study identified tumour-derived galactin-4 and galactin-7 as potential ligands for LILRB3, that result in suppression of TAMs upon binding to LILRB3. These interactions could be blocked by a specific LILRB3 mAb, which retarded tumour growth in galectin-4 expressing MC38 tumour bearing preclinical models [184].

LILRB3 exerts an inhibitory effect on immune effector cells, thus facilitating the evasion of cancer cells from their effects. Notably, LILRB3 expression has also been shown ectopically on some cancer cells, including colorectal cancer cells. High level of LILRB3 expression and decreased CD3^+^/CD8^+^ T cell infiltration was proposed as a strong indicator of poor patient outcomes and resistance to immune checkpoint blockade therapies [196]. This suggests that LILRB3 may confer a mechanism by which tumour cells can subvert immune surveillance and resist therapeutic interventions. It was hypothesized that the engagement of LILRB3 on tumour cells with their ligands initiates inhibitory signalling cascades, thereby enabling immune evasion [196]. Ligation of LILRB3 on human monocytes results in functional suppression of myeloid cells *in vitro* and promotion of tumour growth *in vivo* [197].

In summary, LILRB3 exhibits the potential to modulate immune responses and foster immune escape mechanisms in cancer. Further elucidation of the precise mechanisms underlying LILRB3-mediated immunomodulation is imperative for the development of targeted therapeutic approaches aimed at counteracting tumour progression and enhancing anti-cancer immune responses.

####### LILRB4

LILRB4 (CD85k, ILT3, LIR5, HM18) consists of 2 extracellular Ig-like domains connected to 3 intracellular ITIM domains. While LILRB4 is primarily expressed on myeloid cells, low expression levels have also been reported in some lymphocytes such as B cells and NK cells [198]. LILRB4 strongly suppresses tumour immunity in the TME, and as a result of this suppression, it limits anti-tumour efficacy in solid tumours [199]. Furthermore, LILRB4 ligation modulates the cytokine secretion profile of macrophages and promotes IL-10 secretion by macrophages *in vitro*. It also inhibits the expression of the pro-inflammatory chemokine IL- 8 [200]. Blockade of LILRB4 activates MDSCs and results in activation of T cells *in vitro* [201]. A humanized anti-LILRB4 (IO-202) has been investigated in Phase I clinical trials as monotherapy or in combination with anti-PD-1 and shown to be relatively well tolerated in patients with no dose limiting toxicity [202].

####### LILRB5

LILRB5 (LIR8, CD85c) has 4 extracellular Ig-like domains and 4 ITIM domains. LILRB5 has been relatively less studied among LILRs and its functional role is unclear [203]. Although its expression and role has not been examined extensively, it has been shown that LILRB5 is expressed on peripheral blood isolated NK cells and monocytes from HCC patients [204]. An *in vitro* study with human cord blood-derived mast cells showed that LILRB5 localizes intracellularly in mast cell granules and is secreted in a soluble form after FcεRI following IgE cross-linking and plays a role in mast cell dependent inflammatory responses [205]. In parallel, it has been shown that the expression of LILRB5 on APCs regulates the inflammatory response and that blocking of LILRB5 increases antigen presentation [206]. In light of these observations, LILRB5 could be considered as a potential immunoregulatory target for the treatment of cancer.

##### Modulating macrophage phagocytic capacity

Another way to shift TAMs from pro-tumour to anti-tumour is by promoting the capacity of TAMs to phagocytose tumour cells. The phagocytic capacity of TAMs is regulated by both activating (“eat me”, e.g. FcγRIIIA) and inhibitory (“do not eat me”, e.g. CD47) signals (**Fig. 3**). CD47, a ubiquitous glycoprotein, that regulates cell migration, axon extension, cytokine production and T cell activation, serves as a critical inhibitory signal, suppressing phagocytosis by binding to the signal-regulatory protein-α (SIRPα) on the surface of macrophages [207]. Notably, several preclinical studies in xenograft mouse models have demonstrated that CD47 blockade represents an effective strategy for cancer immunotherapy, as it enables phagocytosis and killing of tumour cells by TAMs [208, 209]. Thus, the clinical efficacy of CD47 mAbs is being examined in several clinical trials [210]. However, it should be noted that it has been reported that blocking the CD47/SIRPα axis may be ineffective in hypoxic colorectal cancer, as the expression of CD47 showed a negative association with hypoxia [211]. Indeed, despite the widely recognized importance of hypoxia in oncology, understanding the many complex interactions of hypoxia and the TME with cancer biology and therapy remains a work in progress. A thorough understanding of the intricate mechanisms underlying the hypoxia-mediated regulation of TME will facilitate identification of new therapeutic targets. A number of key “eat me” targets are discussed below.

###### SIRPα

SIRPα is a transmembrane protein containing three Ig-like extracellular domains connected with 2 ITIM domains intracellularly [212]. SIRPα is predominantly expressed in neurons, DCs and macrophages. SIRPα is highly expressed on myeloid cells and plays a role in the regulation of the immune system as well as cell migration and phagocytic activity through its interaction with CD47 [213]. CD47 expression on host cells interacts with SIRPα on myeloid-derived immune cells to deliver “don’t eat me” signals. High CD47 expression on cancer cells provides a strong “don’t eat me” signal by interacting with SIRPα on the surface of myeloid cells. There are several blocking antibodies or fusion proteins developed to disrupt the CD47-SIRPα interaction [214–218]. Nevertheless, it should be noted that the expression of CD47 is not exclusive to tumour cells, and inhibiting CD47 systemically may result in certain toxicities that need to be considered. Indeed, a Phase 3 clinical trial of magrolimab was recently discontinued [219]. The toxicities seen with the CD47 antibodies used initially have led to the ^development of several variations, such as Fc mutated, F(ab’)^2^, scFv, or the fusion of soluble^ SIRPα dimers with human IgG [13, 220–222]. In contrast to CD47, which is widely expressed, the myeloid restricted expression of SIRPα makes it more convenient target. An ongoing human Phase 1 clinical trial, using the SIRPα-directed mAb (BYON4228) in combination with rituximab, has recently begun (see **Table 3** for a list of clinical trials) [223].

**Table 3.** A summary of notable past and current clinical trials investigating the therapeutic targeting of the SIRPα/CD47 axis.

Agent	Mechanism	Completed Clinical Trials Identifiers	Ongoing Clinical Trials Identifiers	Indication	Recommended usage
TTI-621 (SIRPαFc)	SIRPα- human IgG1 Fc soluble fusion protein, designed to block the SIRPα/CD4 7 axis and promote ADCP.	NCT02663518 (phase I; “study terminated due to administrative reasons” NCT02890368 (phase I; terminated; no results published) NCT04996004 (phase I/II; “study terminated due to administrative reasons”)	NCT05507541 (phase II; recruiting)	Relapsed or refractory haematologic malignancies and solid tumours.	Combination with pembrolizumab “may kill more cancer cells in patients with relapsed/refractory diffuse large B- cell lymphoma”.
Hu5F9-G4 (Magrolimab )	Humanized Fc silent CD47 mAb that inhibits the CD47- SIRPα interaction, enhancing ADCP	NCT02678338 (phase I; completed; “Hu5F9-G4 treatment resulted in a haemoglobin decline and increased transfusion requirements.” ) NCT05079230 (phase III; “study terminated due to futility.”) Other 8 clinical trials completed, 13	NCT04788043 (phase II; active, not recruiting) NCT04435691 (phase Ib/II; active, not recruiting) NCT03869190 (phase Ib/II; active, not recruiting) With other 2 phase I clinical trials also active but not recruiting.	Relapsed or refractory haematologic malignancies and advanced solid tumours.	Combination with pembrolizumab. Giving magrolimab, azacitidine, and venetoclax may help to control acute myeloid leukaemia.
		terminated and 6 trials withdrawn. Terminations were mainly due to sponsor decision.			
CC-90002	CD47 mAb aimed at blocking the CD47- SIRPα interaction to promote anti-tumour immunity.	NCT02641002 (phase I; terminated as “the preliminary monotherapy data did not show encouraging results.”) NCT02367196 (phase I; completed; no results published)	None	Acute myeloid leukaemia (AML), high-risk myelodysplastic syndromes (MDS), and haematological neoplasms.	As a single agent or in combination with other cancer therapeutic modalities.
ALX148	High-affinity SIRPα variant fused to an inactive Fc region, designed to block CD47 and enhance immune cell activation.	NCT04755244 (phase I/phase II; terminated; “study never moved forward to phase II”)	NCT03013218 (phase I; active, not recruiting) With several other clinical trials (15 total) in phase I/phase II recruiting/activ e.	Advanced solid tumours, non- Hodgkin lymphoma (NHL), and AML.	Trials generally focussed on using it in combination with chemotherapy or immune checkpoint blockade.
BYON4228	Humanized SIRPα mAb to block SIRPα to CD47 and inhibit signalling through the CD47/SIRP α axis	None	NCT05737628 (phase I; recruiting)	Relapsed/Refractory CD20 Positive B- cell Non-Hodgkin’s Lymphoma (NHL).	Considering using it alone or in combination with rituximab.

###### Sialic acid-binding Ig-like lectin 10 (Siglec-10)

Siglec-10 is an inhibitory receptor expressed on various immune cells, including B cells, monocytes, DCs, and a small number of NK and activated T cells. Siglec-10 has 5 extracellular Ig-like domains, a transmembrane region and 2 ITIMs in its cytoplasmic tail [224]. CD24 is an additional “don’t eat me” signal that interacts with Siglec-10 on innate immune cells and thus inhibits inflammatory responses [225]. CD24 is significantly upregulated in several solid tumours, with the highest increase in ovarian cancer and TNBC compared to healthy or ER^+^PR^+^ breast cells. CD24 expression is negatively correlated with relapse-free and overall survival in ovarian and breast cancer patients [226]. Genetic deletion and therapeutic inhibition of either CD24 or Siglec-10, as well as blocking their interaction using mAbs, can augment the phagocytic capacity of macrophages in CD24-expressing human tumours and promotes tumour-specific responses [225, 227]. High level of Siglec-10 expression on HCC TAMs was correlated with impaired CD8^+^ cells, which was reverted by blocking of Siglec-10 [228]. The CD24/Siglec-10 axis is therefore a promising target for cancer immunotherapy, particularly as Siglec-10 is highly expressed on TAMs and promotes immune evasion [226]. A recent ongoing clinical trial is investigating the therapeutic potential of a humanized antibody targeting Siglec- 10 (NCT06352359) in metastatic solid tumours.

###### Fc gamma RIIB (FcγRIIB)

FcγRIIB, also known as CD32B, is the only inhibitory member of the FcγR family [229]. FcγRIIB is a single-chain, low-affinity receptor for the Fc portion of IgG. It has a molecular weight of ~40 kDa and comprises a single intracellular ITIM domain linked to a transmembrane domain and 2 extracellular Ig-like domains [230]. FcγRIIB plays its immunosuppressive role both via intracellular ITIM domain signalling and competition with activating FcγRs [231]. FcγRIIB is expressed on B cells and myeloid cells as well as epithelial and sinusoidal cells at varying levels depending on cell subtype and differentiation status. In addition to healthy cells, FcγRIIB is expressed on the surface of B cell leukaemia/lymphoma cells [232].

In the last decade, our understanding of the role of FcγRIIB in the formation of an immunosuppressive TME and its potential for enhancing the effectiveness of antibodies used in cancer treatment has grown substantially. FcγRIIB is highly upregulated on TAMs in hypoxic TME, rendering them immunosuppressive [145]. It also plays a regulatory role in the differentiated MDSCs within tumours [233]. Antibodies directed against HER2 in breast cancer gain more effective Fc-dependent effector function by reducing their affinity for the inhibitory FcγRIIB through Fc engineering [234]. Moreover, the phagocytic function of macrophages can be restored by blocking FcγRIIB [145]. For instance, in preclinical models, Tregs are more efficiently depleted when anti-CTLA4 is combined with FcγRIIB blocking antibodies, which potentiate the phagocytic potential of TAMs [235]. Similarly, the therapeutic efficacy of anti-PD-1 has been shown to be reduced due to Fc-mediated trogocytosis of the PD- 1 mAb from the surface of T cells by TAMs, and blocking FcγRII and FcγRIII overcomes this resistance in preclinical models [236]. Collectively, these observations suggest therapeutic targeting of FcγRIIB is likely to potentiate the treatment of a wide range of cancers, particularly when combined with other modalities. An ongoing clinical trial focuses on overcoming resistance to immune checkpoint inhibitors in various solid tumours by a mAb targeting FcγRIIB (BI-1206) in patients with advanced solid tumours who were previously treated with anti-PD-1 or anti-PD-L1 (NCT04219254). Another clinical trial is investigating the effects of blocking FcγRIIB (with an Fc-null mAb, lacking effector functions; BI1607 [231]) in combination with trastuzumab in HER2^+^ advanced solid tumours (NCT05555251), with encouraging interim results to-date. These two first-in-class mAbs, which specifically target human FcγRIIB, have already demonstrated clinical benefit in a number of patients and therefore, may gain regulatory approval in the coming years.

### Concluding remarks

Cancer immunology and particularly T cell checkpoint inhibitors have revolutionized the way cancer patients are treated, with many new immunotherapeutics approved in the last decade. However, a considerable number of patients fail to respond to such treatments or develop resistance over time [237–239]. Hence, further knowledge of the heterogeneity within tumours and identification of novel targets is essential for predicting treatment outcomes and designing new therapies. Due to their high prevalence in the TME and their potent immunoregulatory functions, TAMs present an attractive target and have attracted a significant amount of interest in the field, with many biotechnology and pharmaceutical companies focussing in this area. However, to develop effective TAM-targeted therapies, robust models representing human tumours and the immune system are required. Although syngeneic mouse models are informative, they often fail to faithfully model cancer patients and/or do not express the equivalent targets (e.g. mice do not have LILRB homologues). To overcome such barriers, mice reconstituted with human immune system (‘humanized’) and engrafted with patient-derived or cell-derived xenografts can be engineered [240, 241], that allow testing of the efficacy and safety of experimental drugs, including those targeting TAMs. Additionally, advances in generating human tumour organoids have enabled high throughput testing of drug candidates with high precision [242]. By understanding the complex interplay between TAMs and the TME, researchers can develop novel therapeutic approaches that improve patient outcomes.

Importantly, when designing therapeutic mAbs, a detailed understanding of the antibody structure and mechanism of action is critical in choosing the optimal epitope and isotype [243–246]. Additionally, further modifications of the antibody Fc may be required in order to achieve the desired outcome and prevent clinical trial failures. For instance, an intact Fc is required for optimal anti-PD-L1 activity *in vivo* [247, 248], whereas, in the case of blocking FcγRIIB on myeloid cells, an Fc-null mAb (i.e. unable to engage any FcγRs) is desirable [231]. More technological developments, such as generation of chimeric antigen receptor expressing monocytes or macrophages [249, 250], and nanotechnology-based [251, 252] or extracellular vesicle-based [253] drug delivery systems [251, 252] are expected to pave the way for the next generation of cancer therapeutics.

In summary, preclinical and clinical studies have shown that targeting TAMs can enhance the efficacy of immune checkpoint inhibitors in several cancer types. Therefore, targeting and modulating TAMs represents a promising strategy to address unmet needs in refractory cancers, particularly in enhancing the efficacy of existing checkpoint inhibitors and conventional treatments. It is anticipated that a significant number of TAM-targeted therapies will receive regulatory approval in the near future. These therapies are expected to complement existing clinical treatments, leading to an enhancement in the depth and duration of responses, with a particular focus on improving outcomes in patients with refractory and difficult-to-treat malignancies.

## Permission to reproduce

All figures were generated with BioRender.com.

Figure 1: https://biorender.com/v67o359

Figure 2: https://biorender.com/s36p829

Figure 3: https://biorender.com/h79n993

Figure 4: https://biorender.com/a18o606
